# Amphibious Shelter-Builder Oniscidea Species from the New World with Description of a New Subfamily, a New Genus and a New Species from Brazilian Cave (Isopoda, Synocheta, Styloniscidae)

**DOI:** 10.1371/journal.pone.0115021

**Published:** 2015-05-20

**Authors:** Leila A. Souza, Rodrigo L. Ferreira, André R. Senna

**Affiliations:** 1 Universidade Estadual do Ceará (UECE), Campus do Itaperi, Instituto Superior de Ciências Biomédicas, Laboratório de Carcinicultura/LACAR, Fortaleza, CE, Brasil; 2 Universidade Federal de Lavras (UFLA), Departamento de Biologia, Setor de Zoologia, Laboratório de Ecologia Subterrânea, Lavras, MG, Brasil; 3 Universidade Federal da Bahia (UFBA), Instituto de Biologia, Laboratório de Invertebrados Marinhos: Crustacea, Cnidaria & Fauna Associada (LABIMAR), Salvador, BA, Brasil; Université Pierre et Marie Curie, FRANCE

## Abstract

The new subfamily Iuiuniscinae, Styloniscidae, is erected for the new genus *Iuiuniscus* and the new species *I*. *iuiuensis*, which is described from cave of the State of Bahia, Northeastern Brazil. A special ecological character is shown here for the first time for a New World Oniscidea: the construction of mud shelters. An introduction addressing the systematics of Synocheta with emphasis on Styloniscidae Vandel, 1952 is provided, as well as general comments about the dependence of water in some Oniscidea and ecological traits of amphibious Synocheta. The problems referring to nomenclature, taxonomy and the interrelationships in Styloniscidae are discussed.

## Introduction

According to Schmidt [1], the Synocheta Legrand, 1946 comprise about 630 species and they are adapted to endogeous or cave habitats, all of them confined to quite moist environments. Schmalfuss [2] distinguished two phyletic lines in Synocheta: 1. Pleopod 1 endopodite of male is elongated in relation to that of the female, and simple, without any “individualized setae” or other appendages; exopodite shows specializations related to the copulation behavior. 2. Pleopod 1 endopodite of male is elongated and bears an individualized long seta, which in some groups evolved into an apparent second article of this appendage. The male pleopod 1 exopodite is not—or only slightly—modified. This group includes "Trichoniscidae s. str." [1], Styloniscidae Vandel, 1952, as well as Haplophthalminae, *Buddelundiella* Silvestri, 1897, “Turanoniscidae”, Titanidae, Schoebliidae, and several holarctic genera.

The current section Synocheta used to correspond to the family Trichoniscidae Sars, 1899. Smaller families kept being described, increasing the group’s complexity. The monophyly of Synocheta and their relation with Crinocheta as their sister group were established by Schmalfuss [3]. Synocheta, previously named “complexe trichoniscoide” [4,5], were divided by Vandel into two superfamilies—called “series styloniscienne” and “series trichoniscienne” [5] and, later, Styloniscoidea and Trichoniscoidea [6]—defined by the structure of the copulatory apparatus of the male and by the geographical distribution. This division was adopted by some authors [7–10], but ignored by others [3]. Vandel himself [11] viewed the differences between the two groups as “quantitative” and not “qualitative”, because the strong pleopodal musculature of Styloniscidae was supposed to have been reduced in Trichoniscidae, in a scenario of {Styloniscidae → Trichoniscidae} defined by him.

Verhoeff [12] was the first to mention that Trichoniscidae did not occur in the Neotropical Region. In "Landisopoden aus Südamerika", submitted to publication in 1939 and only actually published 12 years later, Verhoeff [13] created the genus *Patagoniscus* with the species *P*. *iheringi*, *P*. *nordenskjoldi* and *P*. *pallidus* from Patagonia and Falkland Islands. In a second article, prepared in the same period but published earlier, on the fauna of Synocheta from Southern Chile [12], the author described three more species of *Patagoniscus*—*P*. *schwabei*, *P*. *araucanicus* and *P*. *simrothi*. According to Verhoeff [13], *Patagoniscus* is similar to *Trichoniscus* Brandt, 1833 in many aspects, but differs mainly in having only four pairs of fully developed pleopods in both sexes and strong musculature in the first pleonal segment of the male. Verhoeff considered these characters essential to justifying making this genus the type of a new family—Patagoniscidae. In this family, he also included many other Trichoniscidae from South America, and concluded that Trichoniscidae was absent in South and Central America and in a large portion of North America, not taking artificially introduced species into account.

Vandel [5] published a study on “Les trichoniscides (crustaces—isopodes) de l’hemisphere austral…” (although this was the title of the paper, the author suggested a new family for these species), confirming the Verhoeff’s hypothesis [13] that the concerned "Trichoniscidae" (Styloniscidae) have musculature in the first pleonal segment of the male different from all species of *Trichoniscus* of the Northern Hemisphere. Thus, Vandel saw reasons to support Verhoeff’s opinion [13] that Trichoniscidae from South America should be separated from the ones of the Northern Hemisphere as a new family. Vandel [5] didn’t examine Verhoeff’s species, but claimed that he must have misinterpreted his material in many aspects. He considered *Patagoniscus* as junior synonym of *Styloniscus* Dana, 1852, and claimed that both genus and type species, *S*. *magellanicus* Dana, 1852, are valid, despite Dana’s incomplete description [14]. He also preferred not to keep Verhoeff’s name, and named this new family Styloniscidae. The family name created by Vandel, though more recent, was kept instead of Patagoniscidae, because it was established before 1961 and received general acceptance according to article 40 of ICZN [10].

Besides Styloniscidae, there are two other small families included by Vandel [5] in Styloniscoidea, both endemic from Southern Africa: Schoebliidae and Titanidae. Their species are exclusively termitary dwellers of depressed body [5]. Information about regressive characters probably connected to the microhabitat of termitaries can be found in Kensley [8]. Borutzky [7], based on a termitary dwelling species of Uzbekistan of which only females are known, described a fourth family in Styloniscoidea—Turanoniscidae.

Schmalfuss [3] didn’t recognize the superfamilies Styloniscoidea and Trichoniscoidea because he didn’t find evidences of their monophyly or of a sister group relation between them. This author included Olibrinidae Budde-Lund, 1913, a small group of halophile Oniscidea, in the infraorder Synocheta, besides the families recognized by Vandel. But Olibrinidae has at least one possible synapomorphy with the Crinocheta—tuft of penicillia (and not teeth) in the molar process of mandible—so that such inclusion would need more convincing backing.

Schmölzer [15] and Tabacaru [16] approached Trichoniscidae systematics, giving formal names to the tribes of Trichoniscinae called “legions” by Vandel [6]. Tabacaru [16] recognized the two traditional subfamilies, Trichoniscinae and Haplophthalminae Verhoeff, 1908. He elevated a tribe poorly described not long before—Thaumatoniscellini—to the rank of subfamily; but this was done in the last line of his article, in a not very explicit way, and was not even referred in the abstract. He also recognized the proximity of Buddellundiellidae Verhoeff, 1930 to Haplophthalminae and Trichoniscinae Trichoniscini, as had already been proposed by Vandel. Neither of the authors, however, passed this phylogenetic idea to their classifications, keeping Trichoniscidae as a concededly paraphyletic group.

According to Schmidt [1]: “the Trichoniscidae are a paraphyletic basal group including all species not having the apomorphies of the other families”.

Classification scheme for the Trichoniscidae of Vandel/Schmölzer/Tabacaru:

**Table:** 

Trichoniscidae Sars, 1899
Trichoniscinae Sars, 1899
Première Division = Typhlotricholigioidini Rioja, 1959
Deuxième Division
Légions I + II = Spelaeonethini Schmölzer, 1965
Légion III = Androniscini Tabacaru, 1993
Légion IV = Oritoniscini Tabacaru, 1993
Légion V = Trichoniscoidini Schmölzer, 1965
Troisième Division = Trichoniscini Sars, 1899
Haplophthalminae Verhoeff, 1908
Thaumatoniscellinae Tabacaru, 1993

The family Styloniscidae was divided by Vandel [5] into two subfamilies: Styloniscinae and Notoniscinae. The first, which seems to be defined only by symplesiomorphies, is characterized by smooth or tuberculated dorsum and pleon-epimera 1–5 reduced opening a gap between pereon and pleon. The Notoniscinae are a small group of species with well-developed neopleurons 3–5 or 4–5 and tergal ornamentation similar to the one found in Haplophthalminae (Trichoniscidae), with tubercles or longitudinal ribs and sometimes pleonites with protrusions. Strouhal [17] described a third subfamily—Kuscheloniscinae—from the Juan Fernández Islands (Chile), monotypic and defined by the absence of an interruption in the pereon-pleon outline, pleon-epimera 3–5 very reduced and by having pereonites with protrusions and lateral ribs.

Until the present study, Styloniscinae was grouped the following genera [18, 19]:

*Clavigeroniscus* Arcangeli, 1930, groups five species (only four, according to Tabacaru [16]) with pantropical distribution. One of them—*C*. *orghidani* Vandel, 1981, from Cuba, is troglobiotic.

*Cordioniscus* Graeve, 1914 (maybe a synonym of *Styloniscus* [20]), includes 15 species. Among them, *C*. *stebbing* (Patience, 1907) is cosmopolitan and *“Cordioniscus” leleupi* Vandel, 1968 is exclusive from Ecuador [21]. Of the remaining species, two are troglobiotic from Bulgaria: *C*. *bulgaricus* Andreev, 1986 and *C*. *schmalfussi* Andreev, 2002 and the rest from mediterranean countries, mainly Greece [22], of which at least six are troglobiotic.

*Indoniscus* Vandel, 1952, presents three species from regions surrounding the Indian Ocean. One of them, *I*. *deharvengi* Dalens, 1987, from Thailand, is troglobiotic.

*Pectenoniscus* Andersson, 1960 is monotypic, from Southern Brazil.

*Trogloniscus* Taiti & Xue, 2012 (includes *Sinoniscus* Schultz, 1995 and *Guiliniscus* Dixon, 2013) has five troglobiotic species from the Chinese provinces of Guangxi and Guizhou (although the largest part of the Chinese territory belongs to the Palearctic Region, these provinces lie in the Oriental Region).

*Styloniscus* Dana, 1852, groups about 40 species of mainly circum-antarctic distribution, some of them troglobiotic.

*Thailandoniscus* Dalens, 1989 is monotypic, with one troglobiotic species from Thailand.

*Spelunconiscus* Campos-Filho, Araújo & Taiti, 2014, monotypic, with one troglobiotic species (*S*. *castroi*) from state of Minas Gerais, Brazil.

*Xangoniscus* Campos-Filho, Araújo & Taiti, 2014, monotypic, with one troglobiotic species (*X*. *aganju*) from state of Bahia, Brazil.

The subfamily Notoniscinae groups two genera, *Notoniscus* Chilton, 1915, from the Australian Region, and *Paranotoniscus* Barnard, 1932, from South Africa, both without regressive characters.

On the other hand, Kuscheloniscinae includes a single monotypic genus, *Kuscheloniscus* Strouhal, 1961, with one species from Southern Neotropics, without regressive characters.

Considering the diagnostic characters pointed to by Vandel [5] and Schmalfuss [2], the new genus and new species described here belong to the family Styloniscidae, but their inclusion in one of the previously established subfamilies is impossible. A comprehensive analysis of Brazilian Styloniscidae, started by Souza-Kury [23] and still in progress, reveals the need for the creation of a new subfamily for these Styloniscidae. Thus, a new subfamily is herein erected based on the new genus and new species herein described. Recently Campos-Filho *et al*. [19] published an article on Brazilian cave terrestrial isopods, in which they described two monotypic genera of Styloniscidae. Campos-Filho *et al*. [19] noted that the taxonomic impediment has been a problem in the description of Oniscidea species. The present paper contributes to solving this problem.

This is the first paper of a series which the authors intend to publish on the Brazilian troglobiotic Isopoda, particularly Styloniscidae, in the BIS Research Group (*Biodiversidade de Isopoda Subterrâneos*) registered in *Conselho Nacional de Desenvolvimento Científico e Tecnológico* (CNPq), Brazil.

## Material and Methods

No specific permissions were required for these locations/activities (it's not a Conservation Unit). However, we have a permission from Instituto Chico Mendes de Conservação da Biodiversidade (ICMBio)—Brazil, because the collection was made in a cave (license nº 14783–1). The field studies did not involve endangered or protected species. It is a new invertebrate species described in this study. Location of the study: “Lapa do Baixão” cave (14º23´8.13´´S, 43º3735.06´´W), located in Iuiu municipality (Bahia state, Brazil).

The specimens were found in “Lapa do Baixão” cave (14º23´8.13´´S, 43º3735.06´´W), located in Iuiu municipality (Bahia state, Brazil). The cave was not totally explored, since part of its inner chambers is flooded during rainy periods. However, the known conduits extend over 500 meters. The only known entrance to the cave is located at the bottom of a subsidence sinkhole, which receives epigean water, especially during the rainy season. The external area is severely impacted, mainly by human activities such as agriculture and extensive breeding of cattle and goats. Fortunately, it seems the cave has not been visited by anyone except the research team.

A visual search for specimens was conducted throughout the base and walls of the “Lapa do Baixão” cave. Special attention was paid to water ponds, which were more common in deeper areas of the cave, although some ponds were also observed near the entrance. Specimens were captured with a fine brush and placed in vials containing ethanol 70%. The specimens were dissected under a Leica stereoscopic microscope and their appendages were mounted in glycerol gel slides. The illustrations were made under an optic microscope, Motic BA-310, with camera lucida, and digitally prepared with CorelDraw X6, with support of a tablet, Wacon Intuos 4. The type material is preserved in ethanol 70% and housed at the Crustacea Collection of *Museu de Zoologia*, *Universidade Federal da Bahia* (UFBA), and the Collection of Subterranean Invertebrates, *Universidade Federal de Lavras* (ISLA). Nomenclature of characters is mainly based on Schmidt [24].

### Nomenclatural Acts

The electronic edition of this article conforms to the requirements of the amended International Code of Zoological Nomenclature, and hence the new names contained herein are available under that Code from the electronic edition of this article. This published work and the nomenclatural acts it contains have been registered in ZooBank, the online registration system for the ICZN. The ZooBank LSIDs (Life Science Identifiers) can be resolved and the associated information viewed through any standard web browser by appending the LSID to the prefix “http://zoobank.org/”. The LSID for this publication is: urn:lsid:zoobank.org:pub:F5155225-9832-41AA-A476-97041BD837B3. The electronic edition of this work was published in a journal with an ISSN, and has been archived and is available from the following digital repositories: PubMed Central, LOCKSS.

## Results

Order Isopoda Latreille, 1817

Section Synocheta Legrand, 1946

Family Styloniscidae Vandel, 1952

Subfamily Iuiuniscinae subfam. nov.

### Etymology

Subfamily name based on genus name.

### Diagnosis

Large, amphibious troglobiotic Styloniscidae about 10 mm long; flexible body capable of folding; dorsal integument smooth or without ribs or large protrusions (*versus* with tubercles or longitudinal ribs in Notoniscinae and *versus* protrusions and lateral ribs in Kuscheloniscinae); enlarged coxal plates; pereopod 1 much shorter than the others, flanking the head; pleon-epimera 3–5 well developed (*versus* pleon-epimera 1–5 reduced in Styloniscinae and *versus* pleon-epimera 3–5 very reduced in Kuscheloniscinae), forming acute tips; telson distal half is lower than the proximal half. This subfamily includes shelter-builder species.

Genus *Iuiuniscus* gen. nov.

### Etymology

Genus name is arbitrary, from Iuiú, name of the type locality + the ending (o)niscus used for oniscideans. Gender masculine.

### Diagnosis

Blind and unpigmented. Amphibious, can be found inside water bodies in the cave. Also in the cave, it can be found in special habitats: semi-spheric clay formations on the walls, or natural holes sealed with clay, where it hides. Broad enlarged and acute coxal plates; pleon-epimera 3–5 with exceptionally long and acute tips. First antenna with ten aesthetascs. First maxilla outer endite with 4+3 simple tooth setae, one slender seta in the inner corner and two long bipectinate setae in the inner group of teeth. Telson with acute apex. Second antennae have a 7-jointed flagellum. Pereopods 2–4 of male with stout protrusion proximally on the frontal face of the merus.

Type species. *Iuiuniscus iuiuensis* gen. *et* sp. nov., by monotypy.

**Fig 1:**
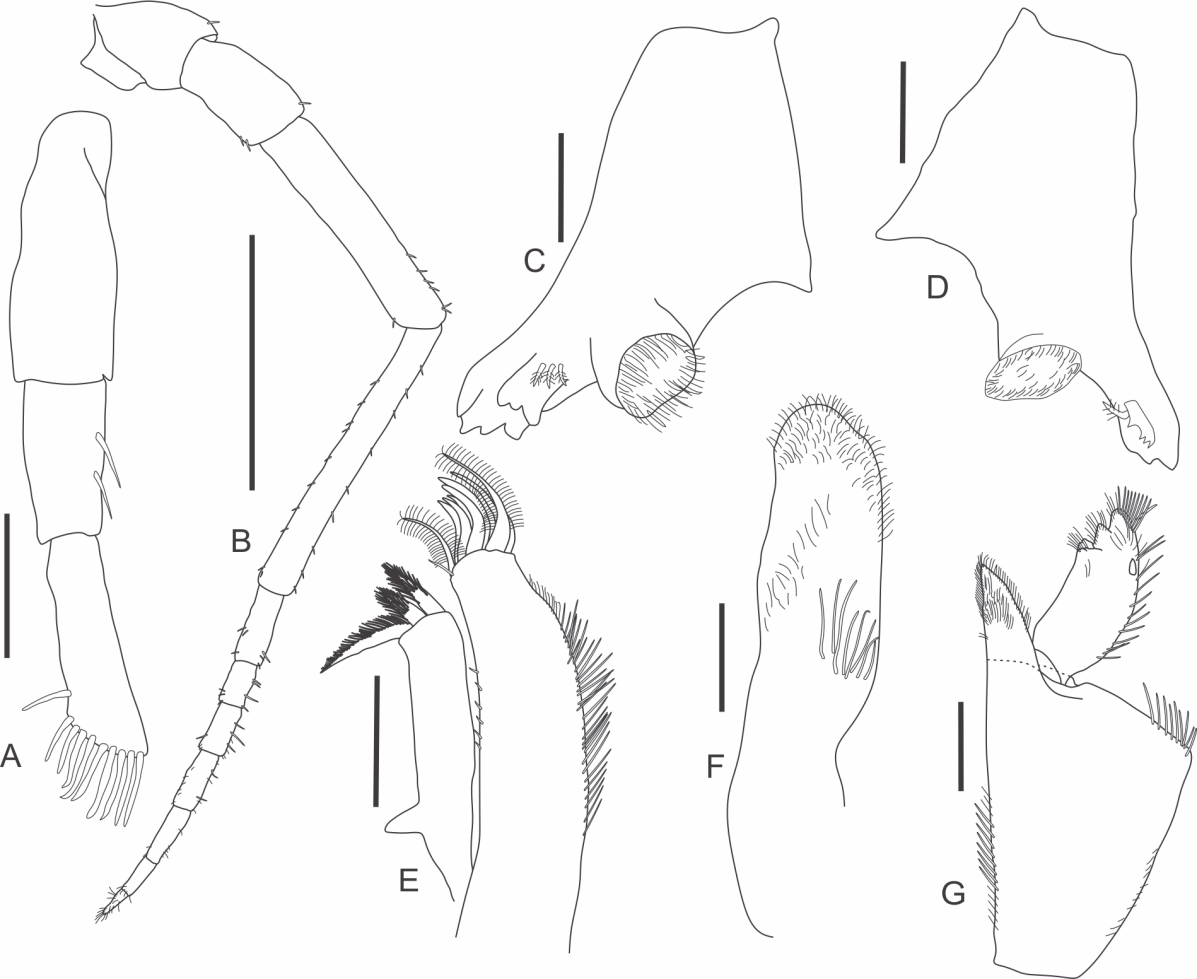
*Iuiuniscus iuiuensis* gen. *et* sp. nov. (Holotype, UFBA 1603). (A) First antenna, (B) Second antenna, (C) Left mandible, (D) Right mandible, (E) First maxilla, (F) Second maxilla, (G) Maxilliped. Scale bars: 0.1 mm for A; 1.0 mm for B; 0.2 mm for the remainder.

**Fig 2:**
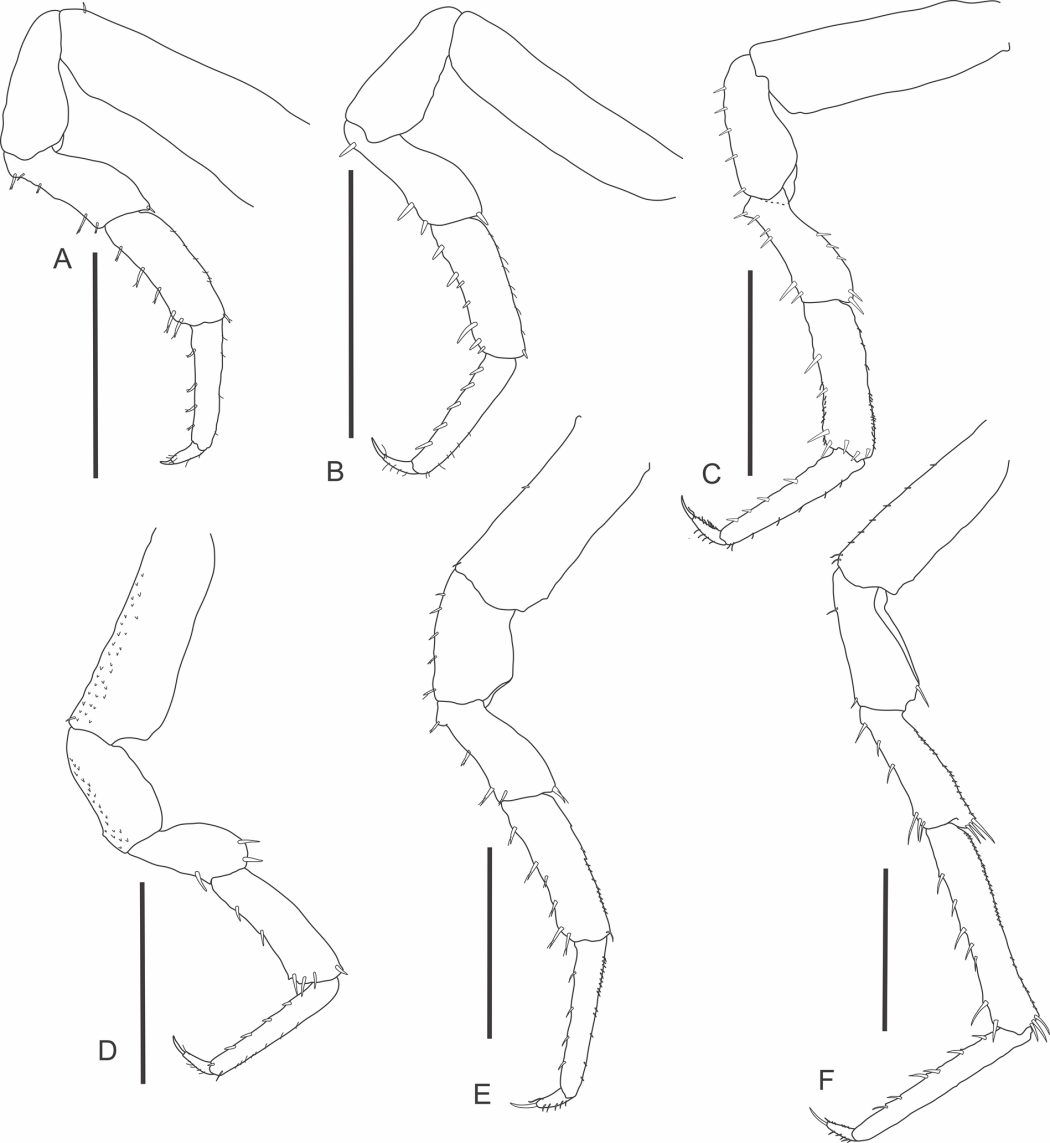
*Iuiuniscus iuiuensis* gen. *et* sp. nov. (Holotype, UFBA 1603). (A) Pereopod 1, (C) Pereopod 2, (E) Pereopod 3, (F) Pereopod 7. (Paratype, female, UFBA 1604). (B) Pereopod 1, (D) Pereopod 2. Scale bars: 1.0 mm.

**Fig 3:**
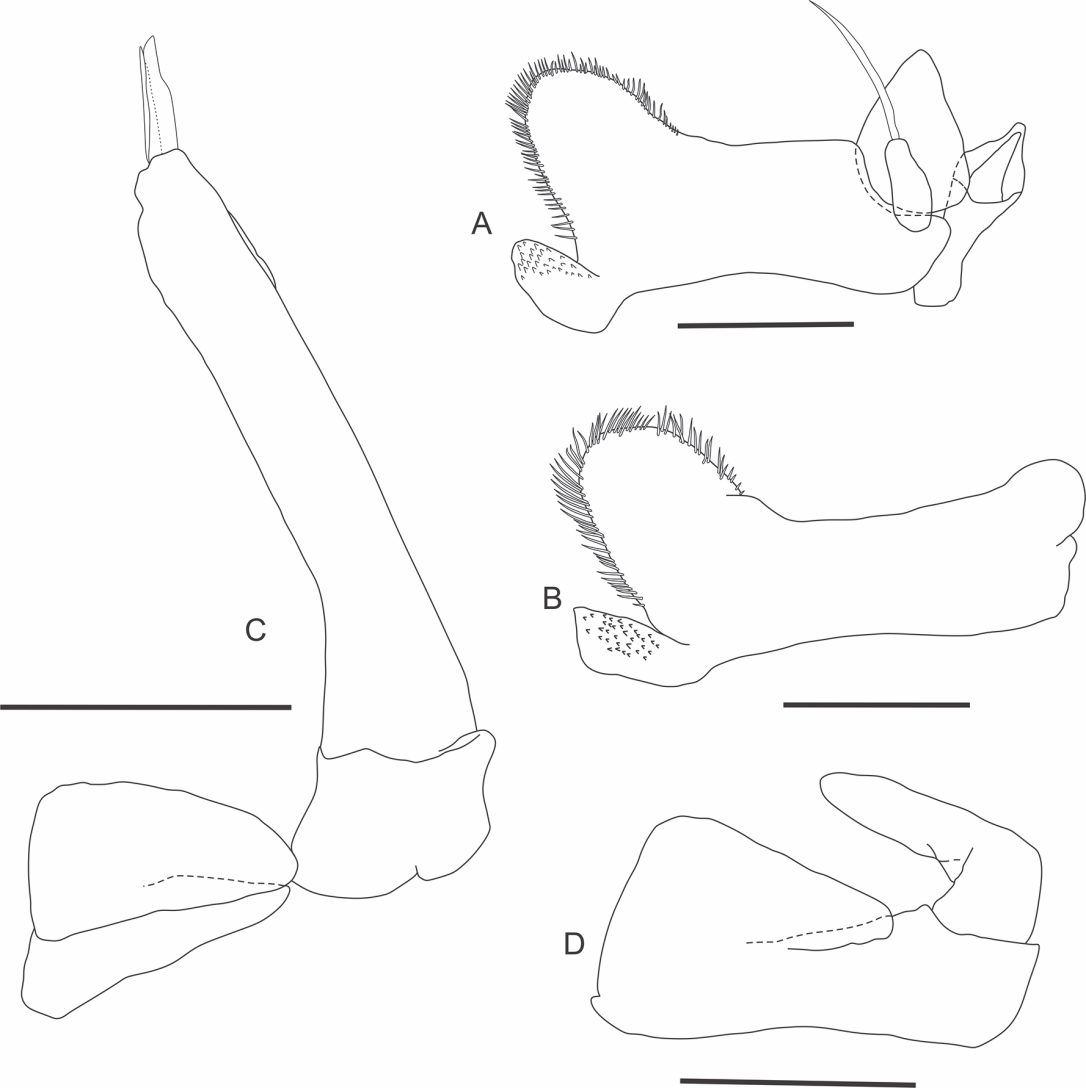
*Iuiuniscus iuiuensis* gen. *et* sp. nov. (Holotype, UFBA 1603). (A) Pleopod 1, (C) Pleopod 2. (Paratype, female, UFBA 1604). (B) Pleopod 1, (D) Pleopod 2. Scale bars: 0.5 mm.

Iuiuniscus iuiuensis *gen*. et *sp*. *nov*.

urn:lsid:zoobank.org:act:D5A719D3-51BE-4D85-AB2A-93A011F58EFD (Figs 1–3)

### Material examined

Holotype: male, dissected and drawn, Lapa do Baixão, Iuiú, BA, Brazil, 20.VII.2007, R. L. Ferreira col., UFBA 1603.

Paratypes: 1 female, dissected and drawn, Lapa do Baixão, Iuiú, BA, Brazil, 20.VII.2007, R. L. Ferreira col., UFBA 1604; 7 males and 1 female, Lapa do Baixão, Iuiú, BA, Brazil, 20.VII.2007, R. L. Ferreira col., UFBA 1605; 3 males and 1 female, Lapa do Baixão, Iuiú, BA, Brazil, 20.VII.2007, R. L. Ferreira col., ISLA 4993.

### Etymology

The species name is an adjective derived from the type locality name, Iuiú.

### Diagnosis

Same as the genus.

### Description

Based in the holotype. Measurements: length—9.2 mm from anterior margin of the head to the apex of telson; width—5 mm, from one tip to another of the pereon coxal plate 4. Cephalon semiquadrangular, fully inserted in the first pereonite; there is a line on the vertex which runs along the back of the posterior deep furrows which hold the second antennae, forming a "V" whose tip is positioned medially between the insertion of the antennae. Without eyes and pigment, with convex body. Dorsal integument smooth, not very thin, with setae and more sclerotized on the tips of coxal plates that are all fused with tergites; posterior margins of tergites of pereonites with different kinds of setae. Flexible body capable of folding. Convex pereon tergites, coxal plates of pereonites 1–7 outwardly extended and widely separated, apically acute. Pereonite 1 is of smaller width, but of greater length, coxal plates go down and then fold up and form a concave area in the anterior end; coxal plates of pereonites 1–7 form lateral-distal ends that become progressively more acute, and are especially developed and acute from pereonite 5–7. Sternum of pereonites deeply grooved at the sites of insertion of pereopods (minor groove is at the insertion of pereopod 1). Pleonites 1 and 2 distinct in dorsal view, without apparent pleon-epimera. Pleon-epimera 3–5 with exceptionally long tips; the tips of the fifth are longer than exopods of uropods. Telson distal half depressed in relation to the proximal half, lateral margin slightly sinuous, with acute apex.

First antenna (Fig 1A) 3-jointed with nine apical and one lateral aesthetascs on the third article; the three articles are subequal in length. Second antenna (Fig 1B) thin; when stretched, it reaches the edge of coxal plate of the pereonite 3; the last article of peduncle is the largest; flagellum with 7 articles, the first one the biggest, the apical one the smallest, with a tuft of free sensory hairs. Mandibles with a pars molaris with a large masticatory surface; left mandible (Fig 1C) with three slender penicils between the pars incisiva and the pars molaris, *lacinia mobilis* robust and apically 3-cuspidate; right mandible (Fig 1D) with one slender penicil between the pars incisiva and the pars molaris, *lacinia mobilis* slender and bipectinate. First maxilla (Fig 1E) inner endite with three penicils, two of them are almost equal and the third is the biggest; without laterodistal corner; outer endite with outer group with four strong and simple tooth setae; inner group with three simple tooth setae + one slender and differentiated seta in the inner corner; there are also two long setae wholly and conspicuously bipectinate, that emerge from the inner group of teeth. Second maxilla (Fig 1F) apically rounded, with many sensilla and outer lobe not apparent. Maxilliped (Fig 1G) with a large basis narrowed in its proximal portion; maxilliped palp with 3 indistinctly delimited distal articles, each of them with a tuft of setae, there is a single setae located below the proximal article; endite distally acute, hairy.

Pereopods 1–3 (Fig 2A–2E) pointing to the proximal region of the body; pereopods 4–7 (Fig 2F), longer, to the distal; spines on margins of pereopods with an accessory seta each. Pereopod 1 much shorter than the others, flanking the head; dactylus with an outer claw big but slender, with a brownish tip indicating heavy sclerotization. Merus of pereopods 2–4 with protrusion at its frontal face, proximally; merus 6 with dilated and truncated distal part. Dactylus of pereopods 1–7 without conspicuous inner claw. Genital papilla (Fig 3A) swollen in the medial part, with extremity constituted of a basal region with folds and a long tip. Pleopod 1 (Fig 3A) sympodite large; endopodite biarticulate, 2nd article flagelliform, tiny, slender, barely visible *in situ*. Pleopod 2 (Fig 3B) exopodite without marginal setae; endopodite biarticulate, enlarged, bifurcate at the apex, forming two long tips. Exopodites of pleopods 1 and 2 smaller and 3–5 bigger, but among the latter, the fifth is the smallest; they all close like blades over the endopods. Sympodites of uropods small. Endopodites of uropods about 2/3 the length of exopodites.

### Sexual dimorphism

Dactylus of pereopod 1 of male with very small and acute inner claw; inner claw of dactylus of pereopod 1 of female bigger. Female pleopod 1 sympodite (Fig 3B), with setae surrounding the entire outer margin of its outer part, which is large and oval, continuing as a rectangular piece that joins the exopodite to form a single piece. In the junction of female pleopod 1 exopodites, in the midline, they form two small lobes at a height of half their length and distally form two larger lobes; endopodites weren’t observed in 2 female specimens dissected.

### Remarks

In the *sein* of the Styloniscidae, the new subfamily Iuiuniscinae is distiguished from Styloniscinae by very developed pleon-epimera 3–5; from Notoniscinae by not having longitudinal ribs; and from Kuscheolinicinae by very developed pleon-epimera 3–5 and by not having protrusions and ribs on the lateral parts of the pereon. It can be distinguished from Notoniscinae and Kuscheolinicinae by being larger and by including only troglobiotic species (Styloniscinae contains genera with troglobiotic species and *Styloniscus* includes species of up to 14 mm), as shall be confirmed in the coming papers on the description of new genera and species. It can be distinguished from all other Styloniscidae subfamilies by all the other characteristics mentioned in the subfamily diagnosis herein provided. Some of these characteristics are synapomorphies (e. g. enlarged coxal plates; pleon-epimera 3–5 well developed). Campos-Filho *et al*. [19] include the two monotypic genera they described, *Spelunconiscus* and *Xangoniscus*, in Styloniscinae. In the diagnosis for *Spelunconiscus*, they mark the main characteristic that discriminates Styloniscinae from the other subfamilies (“pleonites 3–5 with epimera reduced, adpressed, without visible posterior points.”). Conversely, in the diagnosis of the genus *Xangoniscus*, these authors refer to pleonites 3–5 as showing “epimera well developed, with visible posterior points.” Considering the description of this character state for *X*. *aganju* (“Pleonites 3–5 (Fig 9F) with falciform epimera.”) and the corresponding illustration, the insertion of this genus in Styloniscinae remains appropriate, since small variations in the poorly-developed pleon-epimera 1–5 may be interpreted as the same state, which is plesiomorphic (see some comments on the interrelationships in Styloniscidae in the Discussion below). In Styloniscidae, the morphology of the distal part of pleopod 2 endopodite of male has been a character of interest for analysis. Campos-Filho *et al*. [19] point to the resemblance between *Xangoniscus*, *Clavigeroniscus* and *Thailandoniscus* and their difference from *Spelunconiscus* in relation to this character. Souza-Kury [23] had already shown this resemblance between *Clavigeroniscus* and *Thailandoniscus*, and separated this character in two. Considering the morphology of the distal part of pleopod 2 endopodite of male, *Iuiuniscus iuiuensis* gen. *et* sp. nov. is in part similar to *Spelunconiscus*, but not to *Xangoniscus*. *Iuiuniscus iuiuensis* gen. *et* sp. nov. and *Xangoniscus aganju* occur in caves in different cities of the Brazilian state of Bahia.

## Natural History

**Fig 4:**
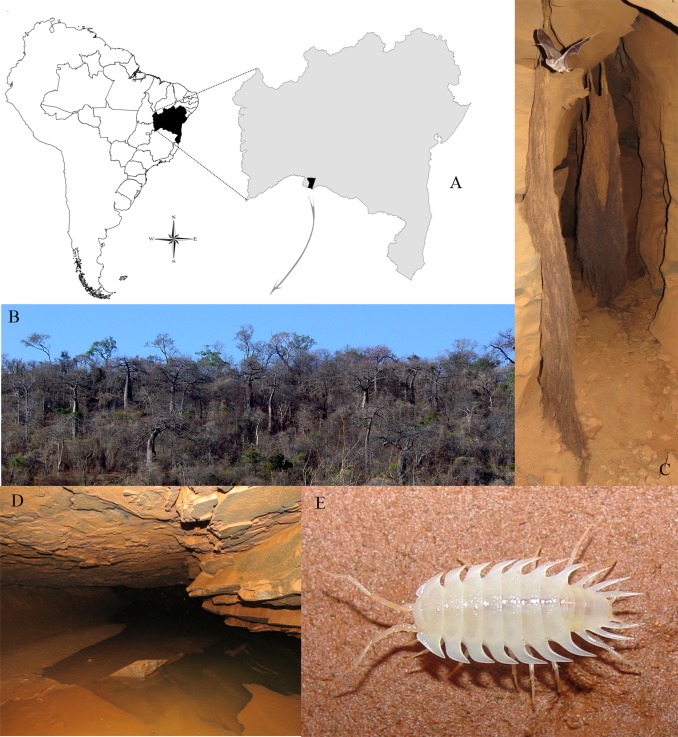
*Iuiuniscus iuiuensis* gen. *et* sp. nov. (A) Iuiu municipality (Bahia state, Brazil), (B) Surroundings of Lapa do Baixão cave, (C) Inside the cave, showing roots, (D) Puddles where specimens were collected, (E) Habitus.

**Fig 5:**
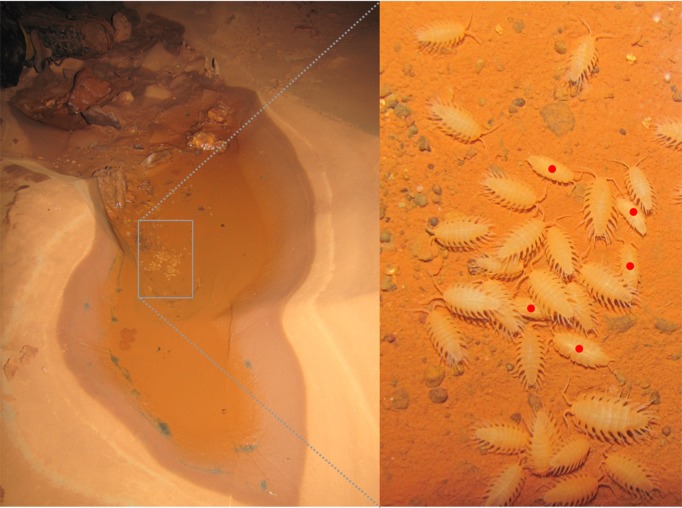
*Iuiuniscus iuiuensis* gen. *et* sp. nov. cohabiting puddle with another species. Red point: sympatric species.

Specimens of *I*. *iuiuensis* gen. *et* sp. nov. were only found inside Lapa do Baixão cave, Iuiu municipality (Bahia state, Brazil) (Fig 4A), although other caves have been sampled in the region of the Caatinga, the only xeric biome of the country with xeromorph, decidual vegetation (Fig 4B). Our research team went to eight other caves near Lapa do Baixão cave, and found no specimens of *I*. *iuiuensis* gen. *et* sp. nov. what indicates that the species could be endemic to this cave. Many roots were observed inside the cave, an important nourishment source to the hypogean fauna (Fig 4C). The organisms were observed mostly inside water bodies, particularly in places far from the entrance. The cave has a water table level that oscillates over the year (water stains of different levels were found on the walls). The fluctuation of the phreatic level produces puddles that teem with specimens. Few were observed directly in the phreatic level, in comparison to the dense groups found in puddles. However, the small volume of the puddles must have led to the specimens being crowded in small areas (which doesn’t happen in the phreatic level, which encompasses a great flooded conduit) (Fig 4D). *Iuiuniscus iuiuensis* gen. *et* sp. nov. cohabits puddles with specimens of another species of Styloniscidae, also troglobiotic (Fig 5). This sympatric species, which will be described in a future paper, doesn’t have the big acute 3–5 pleon-epimera typical of *I*. *iuiuensis* gen. *et* sp. nov. It dwells mainly in areas near the entrance, and wasn’t observed in the deeper regions (including the phreatic level), unlike *I*. *iuiuensis* gen. *et* sp. nov. Although a large part has been observed in puddles, some specimens of both species were found walking on the moist substratum of the cave, both on the ground and on the walls. This clearly demonstrates that they are capable of dispersing when the puddles dry out.

**Fig 6:**
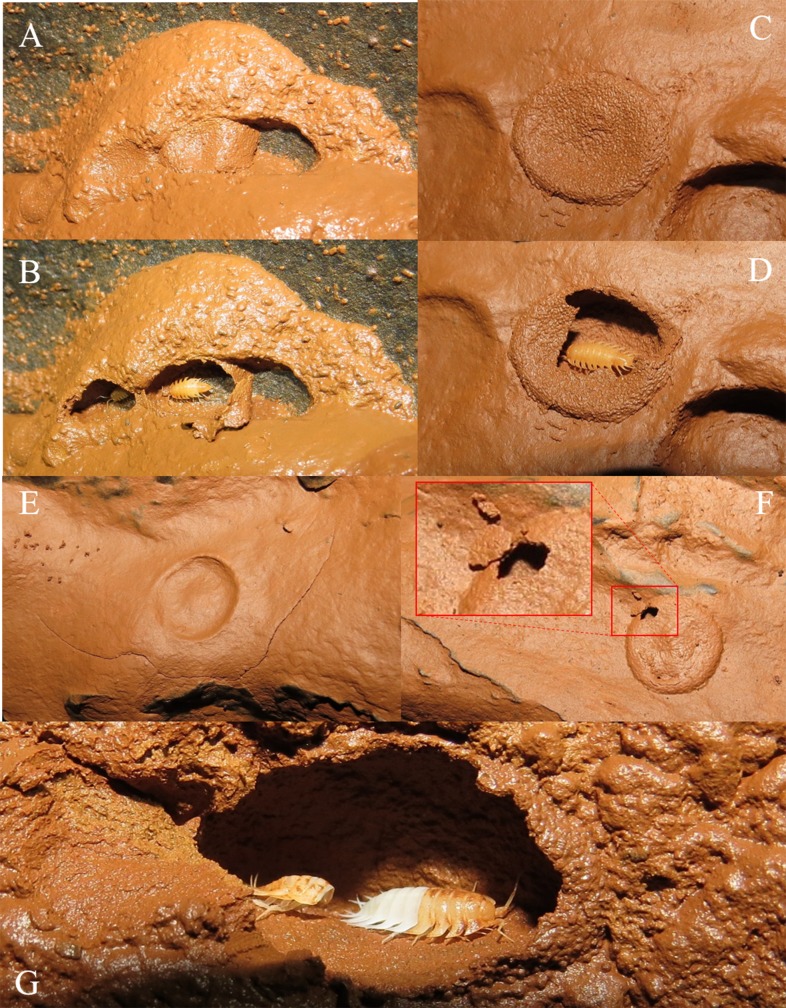
Semispherical and irregular shelters made of clay, built by specimens of *I*. *iuiuensis* gen. *et* sp. nov., and used by them as molting chambers.

*I*. *iuiuensis* gen. *et* sp. nov. shows a special ecological character recorded for the first time in Styloniscidae species. In several areas of the innermost part of the cave, semispherical structures made of clay (Fig 6C) were observed on the walls. Such structures sometimes possessed irregular shapes (Fig 6A). Some of them were opened, revealing specimens of *I*. *iuiuensis* gen. *et* sp. nov. inside (Fig 6B and 6D). The specimens use the clay present on the floor for building the shelter’s walls, since old eroded shelters (without the covering) showed a depression from where the clay used on the walls was extracted (Fig 6E). The specimens also use natural holes in the rock as shelters, using clay for sealing these cavities (Fig 6A and 6B). Among millipedes (Diplopoda), special molting chambers are usually constructed, differing from one order to another [25]. Similarly, the clay structures built by *I*. *iuiuensis* gen. *et* sp. nov. are also molting chambers, used as shelter during the process, when the specimens are vulnerable. The molting specimens of Oniscidea consume the exuvia to obtain its minerals [26, 27] and some not yet consumed exuvias were observed within the chambers, near the specimens under molting (Fig 6G). After the hardening of the new exoskeleton, the specimens leave the shelter by a small opening made by themselves (Fig 6F). The behavior of building shelters for molting could be related to: *i)* desiccation avoidance or *ii)* predation avoidance. The fact that the chambers aim at protecting the specimens from drying is unlikely, because they’re located in very humid areas. However, in the terrestrial habitats of this cave there is a potential predator: a relatively big troglobiotic pseudoscorpion species (*Spelaeobochica iuiu* Ratton, Mahnert & Ferreira, 2012). This species is well distributed along the cave, especially in the areas where *I*. *iuiuensis* gen. *et* sp. nov. occurs. Although this predator cannot offer risks for “regular” specimens, it certainly can offer risks for molting specimens, due to their exoskeleton fragility. Accordingly, the sheltering behavior could prevent their predation during molting.

Among Oniscidea, the behavior of building shelters was observed to serve not only for molting, but also for mating and birth giving. It was mentioned by few authors, in very succinct papers: [28–31]. The “logettes de mue et parturition”, as the shelters were called by the French authors, were recorded for six genera and ten species of Oniscidea—one genus and species of Crinocheta Platyarthridae: *Platyarthrus hoffmannseggii* Brandt, 1833—and five genera and nine species of Synocheta Trichoniscidae: *Metatrichoniscoides fouresi* Vandel, 1950, *Nesiotoniscus corsicus* Racovitza, 1908, *Scotoniscus macromelos* Racovitza, 1908, *Trichoniscoides albidus* (Budde-Lund, 1880), *Trichoniscoides albigensis* Dalens, 1966, *Trichoniscoides modestus* Racovitza, 1908, *Trichoniscoides sarsi* Patience, 1908, *Trichoniscoides vandeli* Dalens, 1966 and *Trichoniscus pygmaeus* Sars, 1898. All species but *P*. *hoffmannseggii*, which is myrmecophilous, are either endogenous or cave-dwelling. Dalens [30] pointed to the fact that the genera *Trichoniscoides*, *Metatrichoniscoides* and *Scotoniscus* are considered to be closely related and to belong to the “Légion V” of Trichoniscinae (Légion V = Trichoniscoidini Schmölzer, 1965, Deuxième Division, see classification above).

Dalens [31] accentuated that two shelters, occupied by one male and one female each, were observed for *Trichoniscoides albidus*, commented the possibility of this fact reflecting the usage of the shelter during copulation, which was confirmed in his paper on *Nesiotoniscus corsicus* [31].

The desert Crinocheta Agnaridae *Hemilepistus reaumuri* (Milne-Edwards, 1840), which lives in burrows, only digs a shelter for survival under harsh conditions, but does not construct any *loget de mue et parturition* [32].

All Oniscidea species that build shelters occur typically in the Palearctic. *Platyarthrus hoffmannseggii*, *Trichoniscoides sarsi* and *Trichoniscus pygmaeus* were introduced in North America [21]. The species herein described is the first New World native cave-dwelling shelter-builder, since none of the Brazilian cave-dwelling species described or recorded in Campos-Filho *et al*. [19] was noted as presenting shelter-building behavior.

Also deserving of mention is the notably differentiated morphology of *I*. *iuiuensis* gen. *et* sp. nov. in comparison to the other Brazilian troglobiotic Styloniscidae. This difference involves the presence of tapering coxal plates of pereonites and pleon-epimera 3–5, so that the habitus could be interpreted as spiny. In no other cave-dwelling species of this family are these tips of pereon and pleon so prominent.

Many troglobiotic Styloniscidae from Brazil dwell in specific microhabitats, especially puddles formed inside dams of travertines. The species that inhabit small streams never occur in places where there are large predators, like fish. However, contrasting to all other know species, *I*. *iuiuensis* gen. *et* sp. nov. inhabits a phreatic level in which fishes of the Trichomycteridae have been observed. This family is quite common in Brazilian caves; 12 out of the 25 Brazilian troglobiotic fishes belong to this family [33].

Morphological modifications, apparently evolved in response to this predation pressure, have already been noticed in other subterranean crustaceans [34]. In the case of some *Troglocaris* species, some species⁄populations consistently co-occurring with their predator, *Proteus anguinus* Laurenti, 1768 (Amphibia: Caudata: Proteidae), have longer rostra than species⁄populations living in a *Proteus*-free locality [34].

Terrestrial isopods have responded to predation pressure by developing a variety of anti-predator strategies, which include a combination of behavioral and morphological traits, as said by Schmalfuss [35].

Although this is still speculation, it’s plausible to consider that the “spines” of *I*. *iuiuensis* gen. *et* sp. nov. have been selected because of predation pressure, different from what occurs with other Brazilian species. These tips probably hinder their being eaten by fish. Besides, the second troglobiotic species of the cave (with no prominent tips) only inhabits areas far from the phreatic level, not in contact with predators. This hypothesis must be tested experimentally.

An aquatic life habit, as found in various species of Synocheta and a few Crinocheta, summarized by Tabacaru [36], is regarded as a secondary condition that evolved several times within the Oniscidea [1].

The first amphibious Synocheta were described by Rioja [37], which called the species *Typhlotricholigioides aquaticus*. The “ligioides” part of the name means *Ligia*-like, not in the morphological sense (as already shown by Schultz [38]), but surely in the aquatic habits. Rioja, based on the observations of the collector, said that the isopods have been found in the remains of puddles, and that this was not accidental, but their normal way of living. Vandel [39] stated that *Titanethes albus* (Koch, 1841), a cave dwelling trichoniscid of the Western Balkan peninsula, lives on the moist clay of the caves, often entering water puddles and passing easily from one milieu to another. Vandel [40] described a second amphibious Synocheta—*Cantabroniscus primitivus*, from caves in Spain—and started a series of papers [41, 42] suggesting that *Typhlotricholigioides* Rioja, 1952 and *Cantabroniscus* Vandel, 1965 were relics of the most primitive Synocheta, still in the amphibious state, and took their existence as a proof of the polyphyly of the terrestrial Isopoda. Other authors, however, did not find evidence that the amphibious habit was a conservation of an ancestral condition, and stated that this could be better explained by secondary acquisition [16, 38].

Karaman [43] cites species from the genera *Trichoniscoides* Sars, 1899, *Brackenridgia* Ulrich, 1902, *Alpioniscus* Racovitza, 1908, *Scotoniscus* Racovitza, 1908, *Bureschia* Verhoeff, 1926, *Cyphonethes* Verhoeff, 1926, *Cretoniscellus* Vandel, 1958 (= *Graeconiscus* Strouhal, 1940) [22, 44], *Mexiconiscus* Schultz, 1964, and *Balearonethes* Dalens, 1977 already referred as amphibious. He adds that he himself knows two undescribed species of *Cyphoniscellus* Verhoeff, 1901 that are also amphibious. All these genera belong to the family Trichoniscidae.

In the family Styloniscidae, only five species, *Thailandoniscus annae* Dalens, 1989, *Trogloniscus clarkei* Taiti & Xue, 2012, *Trogloniscus trilobatus* Taiti & Xue, 2012, *Spelunconiscus castroi* Campos-Filho, Araújo & Taiti, 2014 and *Xangoniscus aganju* Campos-Filho, Araújo & Taiti, 2014 are known to occur in subterranean waters.

## Discussion

A thorough reanalysis of the interrelationships in Synocheta and Styloniscidae is beyond the scope of the present paper, but some comments are due: the Styloniscidae can be considered a monophyletic family only if the highly specialized termitariophilous Titanidae and Schoebliidae are also included. A striking synapomorphy for uniting the three supposed families—the structure of the protopodal musculature of pleopod 1—was recognized long ago. Of the three currently accepted subfamilies, Kuscheloniscinae is monotypic and Notoniscinae is a well defined small group. On the other hand, the nominal subfamily Styloniscinae is based only on negative traits.

The definition of the type species of the genus *Indoniscus* offers some problems. This genus was defined for the species *Trichoniscus mauritiensis* Barnard, 1932, poorly described, which Vandel [45] re-described thoroughly. The author, however, didn’t examine the type material of Barnard and cast some doubts over his own identification of the examined specimens; this suggests that the material might correspond to a new species. Later, Barnard [46] discovered that the specimens of Vandel really didn’t correspond to *T*. *mauritiensis*, and described a new species, *Indoniscus vandeli*, for this material. Taiti & Ferrara [47] examined the type material of *T*. *mauritiensis* and suggested to include the species in *Styloniscus*, providing a few additional illustrations, as well as a discussion in which they defend the view that the only species that should stay in *Indoniscus* is *I*. *vandeli*, while all the other ones should be included in *Styloniscus*. By original designation and by monotypy, the type species of *Indoniscus* must be *T*. *mauritiensis*, in spite of all the diagnosis of *Indoniscus* by Vandel and Taiti & Ferrara being based on *I*. *vandeli*. According to the standard procedure ruled by the ICZN, if any author considers that *I*. *vandeli* (but not *T*. *mauritiensis*) belongs to a genus different from *Styloniscus*, he should describe a new genus to include this species, while *Indoniscus* would become a junior synonym of *Styloniscus*. In our opinion, this procedure would lead to the creation of one more superfluous name, and an alternative would be to formally propose that the International Comission of Zoological Nomenclature use its plenary powers to fix *I*. *vandeli* as type species of *Indoniscus*. This way, in spite of the mistakes (1) of Vandel [45], who didn’t identify the species *T*. *mauritiensis* correctly, and (2) of Taiti & Ferrara [47], who excluded the type species of the genus, keeping its name, the general concept according to the use of the authors would be kept.
